# Molecular Point-of-Care Testing for Respiratory Infections: A Comprehensive Literature Review (2006–2026)

**DOI:** 10.3390/diagnostics16060930

**Published:** 2026-03-20

**Authors:** Ahmed J. Alzahrani

**Affiliations:** 1 College of Medicine, Al-Imam Mohammed Ibn Saud Islamic University, Riyadh 11432, Saudi Arabia; ahmed.j.alzahrani.imam@gmail.com; 2 Laboratory Department, Dr. Sulaiman Al Habib Medical Group, Riyadh 11372, Saudi Arabia

**Keywords:** molecular point-of-care testing, respiratory infections, rapid molecular diagnostics, rapid infectious disease diagnostics

## Abstract

Molecular point-of-care testing (POCT) for respiratory infections has undergone remarkable advancement over the past two decades, driven by technological innovation and urgent clinical needs highlighted by the COVID-19 pandemic. This comprehensive systematic review was conducted following PRISMA 2020 guidelines, synthesizing evidence from 254 peer-reviewed studies published between 2006 and 2026, with detailed analysis of the 30 most relevant papers selected through a rigorous four-stage screening process. The review examines the evolution of molecular POCT technologies, including reverse transcription polymerase chain reaction (RT-PCR), loop-mediated isothermal amplification (LAMP), recombinase polymerase amplification (RPA), and CRISPR-based detection systems. Key findings demonstrate that modern molecular POCT platforms achieve diagnostic performance comparable to laboratory-based testing, with sensitivities ranging from 88% to 100% and specificities from 98% to 100%, while delivering results in 15 to 80 min. These technologies enable rapid, accurate detection of major respiratory pathogens, including SARS-CoV-2, influenza A/B, respiratory syncytial virus (RSV), and atypical bacteria. The integration of microfluidic systems, portable devices, and smartphone-based analysis has expanded access to testing in resource-limited settings, emergency departments, and wearable platforms. This review provides critical insights for clinicians, researchers, and policymakers regarding the current state, clinical applications, and future directions of molecular POCT for respiratory infections.

## 1. Introduction

Respiratory tract infections (RTIs) represent a leading cause of morbidity and mortality worldwide, accounting for millions of healthcare visits and hospitalizations annually. The clinical presentation of RTIs caused by different pathogens—including viruses such as SARS-CoV-2, influenza, respiratory syncytial virus (RSV), and bacteria like Mycoplasma pneumoniae—often overlaps significantly, making accurate differential diagnosis challenging based on symptoms alone. Traditional diagnostic approaches, including viral culture and laboratory-based molecular testing, while accurate, require special equipment, expert staff, and longer times that can delay appropriate treatment decisions and infection control measures.

The COVID-19 pandemic underscored the critical need for rapid, accurate, and accessible point-of-care diagnostic testing. This urgent demand accelerated the development and deployment of molecular point-of-care testing (POCT) technologies that can deliver laboratory-quality results in minutes to hours, directly at or near the patient. Molecular POCT leverages nucleic acid amplification techniques to detect pathogen-specific genetic material with high sensitivity and specificity, enabling timely clinical decision-making, appropriate antimicrobial stewardship, and effective infection control.

This comprehensive literature review examines the landscape of molecular POCT for respiratory infections over the past 20 years (2006–2026), analyzing technological innovations, clinical performance, practical applications, and future directions. This review synthesizes evidence from 254 peer-reviewed studies identified through systematic searches across multiple databases, with a detailed analysis of the 30 most relevant papers that represent the current state of the field.

## 2. Materials and Methods

This systematic review was conducted in accordance with the Preferred Reporting Items for Systematic Reviews and Meta-Analyses (PRISMA) 2020 guidelines to ensure transparency, reproducibility, and methodological rigor. Detailed search methodology and PRISMA compliance documentation are provided in Appendix A.

### 2.1. Literature Search Strategy

A comprehensive literature search was performed across multiple electronic databases, including PubMed/MEDLINE, Scopus, Web of Science, IEEE Xplore, and Google Scholar. The search covered publications from January 2006 to December 2026, encompassing two decades of molecular point-of-care testing development. The search strategy employed a combination of Medical Subject Headings (MeSH) terms and free-text keywords related to molecular diagnostics, point-of-care testing, and respiratory infections.

The primary search string included the following: (“molecular diagnostic*” OR “molecular test*” OR “nucleic acid amplification” OR “RT-PCR” OR “LAMP” OR “isothermal amplification” OR “CRISPR” OR “recombinase polymerase amplification”) AND (“point-of-care” OR “POC” OR “near-patient” OR “bedside” OR “rapid test*”) AND (“respiratory infection*” OR “COVID-19” OR “SARS-CoV-2” OR “influenza” OR “RSV” OR “respiratory pathogen*”). Boolean operators and truncation symbols were adapted to the specific requirements of each database. The reference lists of included studies and relevant review articles were manually screened to identify additional eligible publications.

### 2.2. Study Selection and Eligibility Criteria

Study selection followed a rigorous four-stage process. Initial database searches yielded 1847 records. After removing 613 duplicates using automated tools and manual verification, 1234 unique records underwent title and abstract screening. Two independent reviewers screened titles and abstracts against predefined inclusion criteria, with disagreements resolved through discussion or consultation with a third reviewer. Studies were included if they (1) described molecular diagnostic technologies for respiratory pathogens, (2) involved point-of-care or near-patient testing applications, (3) reported original research, systematic reviews, or comprehensive technical evaluations, and (4) were published in peer-reviewed journals or conference proceedings in English.

Following title and abstract screening, 412 full-text articles were retrieved and assessed for eligibility. Studies were excluded if they focused solely on laboratory-based testing without point-of-care applications, lacked sufficient technical or clinical detail, or were primarily opinion pieces without substantial evidence. This process resulted in 254 relevant studies for data extraction. From this pool, 30 studies were selected for in-depth analysis based on their relevance, methodological quality, citation impact, and contribution to understanding the current state of molecular POCT for respiratory infections.

### 2.3. Data Extraction and Quality Assessment

Data extraction was performed systematically using a standardized form developed specifically for this review. The extracted data included study characteristics (authors, year, country, study design), technology platform details (amplification method, detection principle, target pathogens), diagnostic performance metrics (sensitivity, specificity, limit of detection), operational characteristics (time to result, sample type, user requirements), and clinical validation data. For studies reporting diagnostic accuracy, we extracted true positive, true negative, false positive, and false negative values when available.

An important limitation of this review is the restriction to English-language publications, which may have excluded relevant studies published in other languages, particularly from regions with high burden of respiratory infections such as East Asia, Latin America, and parts of Europe. This language restriction could introduce bias by underrepresenting innovations and clinical experiences from non-English-speaking countries, potentially affecting the generalizability of findings to global contexts. Future reviews should consider multilingual search strategies or collaborations with regional experts to capture the full breadth of international research.

Quality assessment was conducted using appropriate tools based on study design. For diagnostic accuracy studies, we applied the Quality Assessment of Diagnostic Accuracy Studies-2 (QUADAS-2) tool, evaluating risk of bias and applicability across four domains: patient selection, index test, reference standard, and flow and timing. For technology development and validation studies, we assessed methodological rigor, analytical validation, and clinical applicability. Studies with high risk of bias or significant applicability concerns were noted, though not excluded, with limitations discussed in the context of findings.

### 2.4. Data Synthesis and Analysis

Given the heterogeneity in study designs, technologies, and outcome measures, a narrative synthesis approach was employed rather than a meta-analysis. Studies were grouped thematically by technology platform (RT-PCR, LAMP, RPA, CRISPR, and microfluidic systems) and application domain (emergency department, primary care, resource-limited settings, and home testing). Within each category, we synthesized evidence regarding diagnostic performance, operational characteristics, clinical utility, and implementation considerations.

Diagnostic performance metrics were tabulated and compared across platforms where sufficient data were available. For studies reporting sensitivity and specificity with 95% confidence intervals, these were extracted and presented. Where studies reported qualitative or descriptive outcomes, these were synthesized narratively. We critically evaluated the strength of evidence for each technology platform, considering factors such as sample size, reference standard quality, real-world validation, and independent replication. Gaps in the evidence base and areas requiring further research were identified through systematic comparison of available evidence against clinical and operational requirements for point-of-care testing.

## 3. Background and Theoretical Foundations

### 3.1. Principles of Molecular Diagnostics

Molecular diagnostic testing for respiratory pathogens relies on the detection and amplification of pathogen-specific nucleic acids (DNA or RNA) present in clinical specimens. The fundamental principle involves three key steps: (1) nucleic acid extraction from the sample matrix, (2) target amplification through enzymatic reactions, and (3) detection of amplified products through various signaling mechanisms. The sensitivity of molecular methods stems from their ability to amplify minute quantities of target nucleic acids to detectable levels, whereas specificity is achieved through primers and probes designed to recognize unique genetic sequences of target pathogens.

### 3.2. Evolution from Laboratory to Point-of-Care

Traditional molecular diagnostics have been confined to centralized laboratories due to requirements for sophisticated instrumentation, controlled environments, and specialized technical expertise. The evolution toward point-of-care testing has necessitated miniaturization, automation, simplification of workflows, and integration of multiple processing steps into single-use cartridges or chips. Key technological enablers include isothermal amplification methods that eliminate the need for thermal cycling, microfluidic systems that reduce reagent volumes and processing times, and portable detection systems that can operate outside laboratory settings.

### 3.3. Clinical Rationale for Rapid Respiratory Diagnostics

The clinical value of rapid molecular POCT for respiratory infections is multifaceted. First, timely pathogen identification enables targeted antiviral or antibacterial therapy, reducing unnecessary broad-spectrum antibiotic use and associated antimicrobial resistance. Second, rapid diagnosis facilitates appropriate patient triage, isolation, and infection control measures, particularly important for highly transmissible pathogens like influenza and SARS-CoV-2. Third, point-of-care results can reduce emergency department wait times, hospital admissions, and healthcare costs. Fourth, accessible testing in primary care and resource-limited settings can improve disease surveillance and outbreak response capabilities.

## 4. Molecular Testing Technologies and Platforms

### 4.1. RT-PCR-Based Systems

Reverse transcription polymerase chain reaction (RT-PCR) remains the gold standard for molecular detection of respiratory viruses. Several commercial platforms have successfully adapted RT-PCR for point-of-care applications through integration and automation. As summarized in Table 1, RT-PCR platforms demonstrate superior analytical sensitivity but require more complex infrastructure compared to isothermal methods.

The Roche Cobas Liat system represents a prominent example of RT-PCR-based POCT. A systematic review and meta-analysis by Chang et al. evaluated the clinical performance of the Cobas Liat SARS-CoV-2 & influenza A/B assay across 4705 samples from eight studies [1]. The platform demonstrated exceptional diagnostic accuracy with pooled sensitivity of 100.0% (95% CI: 96.7–100.0%) and specificity of 99.7% (95% CI: 98.7–99.9%), with a rapid turnaround time of 20 min [1]. A multicenter study by Robbins et al. further validated this platform across diverse healthcare facilities in the United States, reporting overall percent agreement of 98.8% for SARS-CoV-2 and ≥99.5% for influenza A/B compared to centralized assays [2]. The system’s ease of use and faster time to result compared to laboratory-based testing represent significant advantages for clinical management and transmission reduction [2] (Table 1).

Verbakel et al. evaluated the cobas^®^ Liat^®^ PCR POCT for influenza A/B and RSV detection in primary care settings, testing 744 patients (140 children and 604 adults) [3]. The platform achieved 100% sensitivity for influenza A, influenza B, and RSV, with specificities ranging from 98.1% to 99.7% [3]. Trained technicians found the system easy to use with fast turnaround times, supporting its utility as a decentralized platform for primary care respiratory diagnostics [3].

The Cepheid Xpert platform represents another widely adopted RT-PCR-based POCT system. Abdullah et al. compared the STANDARD M10 assay with Xpert Xpress for rapid molecular diagnosis of SARS-CoV-2, influenza A/B, and RSV across 322 respiratory samples [4]. Both platforms demonstrated comparable high performance with sensitivity and specificity ranging from 98–100% [4]. The STANDARD M10 showed 99.4% overall agreement with Xpert Xpress assays, confirming its suitability for rapid simultaneous detection of multiple respiratory pathogens [4].

Domnich et al. evaluated the STANDARD M10 SARS-CoV-2 assay in point-of-care and critical care settings using 591 samples [5]. The platform reliably detected SARS-CoV-2 in 60 min with sensitivities of 100% for ≥1 gene, 95.5% for ORF1ab, and 99.5% for E gene, alongside 100% specificity [5]. The 100% diagnostic accuracy for nasopharyngeal samples in inactivated transport medium and lower respiratory tract specimens demonstrated their practical utility for rapid molecular diagnosis in acute care settings [5].

Maignan et al. conducted a prospective evaluation of the cobas Liat rapid RT-PCR assay for influenza A/B detection at emergency department triage during the 2017–2018 influenza season [6]. Among 187 patients, triage health care workers performed nasopharyngeal swabs and ran rapid RT-PCR assays, achieving a sensitivity of 0.98 (95% CI: 0.91–1.00) and a specificity of 0.99 (95% CI: 0.94–1.00) [6]. The median time from patient entry to results was 46 min, demonstrating the feasibility and high accuracy of health care worker performed rapid RT-PCR at triage [6].

The Abbott ID NOW™ COVID-19 2.0 assay (Abbott Laboratories, Des Plaines, IL, USA) utilizes isothermal molecular test technology for rapid SARS-CoV-2 detection. Iglesias-Ussel et al. evaluated its clinical performance against three real-time RT-PCR assays (Hologic Panther Fusion, Roche Cobas, and CDC 2019-nCoV RT-PCR) across 3146 evaluable subjects [7]. The platform addresses the time-intensive nature of centralized RT-PCR, providing rapid turnaround for timely diagnosis and infection control, particularly valuable in resource-limited settings [7].

### 4.2. Isothermal Amplification Methods

Isothermal amplification technologies, which amplify nucleic acids at a constant temperature without thermal cycling, have emerged as particularly promising for point-of-care applications owing to their simplicity, speed, and reduced instrumentation requirements.

#### 4.2.1. Loop-Mediated Isothermal Amplification (LAMP)

Loop-mediated isothermal amplification (LAMP) has become one of the most widely adopted isothermal methods for the detection of respiratory pathogens. Jee et al. developed a rapid SARS-CoV-2 RT-LAMP-lateral flow assay (LFA) kit that integrates Chelex-100/boiling nucleic acid extraction with one-step amplification detection [8]. The system achieved 97.8% sensitivity and 100% specificity within 40 min without the need for laboratory instruments, with detection limits of 100 PFU/mL using the simplified extraction method [8]. This field-deployable approach addresses the demand for rapid molecular diagnostics that overcome RT-qPCR’s POCT limitations and rapid antigen tests’ low sensitivity [8].

Mautner et al. developed an RT-LAMP assay for SARS-CoV-2 detection directly from pharyngeal swab samples without prior RNA extraction [9]. The assay amplifies the ORF8 and N genomic regions with high specificity, showing no cross-reactivity when tested against 20 other respiratory pathogens [9]. Notably, the RT-LAMP assay is 12 times faster and 10 times cheaper than routine RT-PCR, making it ideally suited for POCT at airports, railway stations, or hospitals [9].

Mora et al. developed a one-pot RT-LAMP assay for colorimetric detection of SARS-CoV-2 from saliva samples, addressing the challenges of saliva as a sample matrix [10]. The five-step workflow—passive saliva collection, heat treatment at 95 °C for 10 min, addition to RT-LAMP assay, amplification at 65 °C for 40 min, and colorimetric readout—achieved 88% sensitivity and 100% specificity across 127 patient samples, with 98% overall accuracy [10]. This approach demonstrates the potential to improve the clinical utility of saliva for point-of-care testing in mobile clinics and other settings [10].

Garneret et al. developed the “COVIDISC” portable device integrating RNA extraction and RT-LAMP with intercalating dyes or fluorescent probes for SARS-CoV-2 detection [11]. The system achieved sensitivity comparable to RT-qPCR (up to Ct 39, <0.1 TCID50 per mL) and 100% specificity against other human coronaviruses and eight respiratory viruses, delivering results in 20 min to one hour (15 min for high viral loads) [11]. The cost-effective and limited equipment makes it well-suited for rapid on-site detection, enabling quick isolation and reducing logistical burdens [11].

Kshirsagar et al. developed a multiplexed one-pot RT-LAMP test for simultaneous detection of SARS-CoV-2, influenza, and RSV in saliva samples [12]. The system combines RT-LAMP with machine learning-enabled analysis on a battery-powered portable analyzer, achieving area under the curve (AUC) values of 0.82 for RSV, 0.93 for influenza, and 0.96 for SARS-CoV-2 in spiked saliva samples compared to RT-PCR [12]. The elimination of traditional optical components through machine learning makes the system adaptable and cost-effective, expanding testing access in resource-limited settings [12].

Lim et al. developed a point-of-care device based on RT-LAMP that simultaneously detects four respiratory viruses (SARS-CoV-2, influenza A, influenza B, and RSV) plus two controls in less than 30 min without RNA extraction kits [13]. The system includes a disposable microfluidic cartridge with mechanical components for automated sample processing, a low-cost portable optical reader, and a smartphone application for fluorescent image recording and analysis [13]. Validation using swabs spiked with virus particles in nasal fluid demonstrated accurate detection with the ability to deconvolve coinfection information [13].

Ruili et al. developed an integrated microsystem based on real-time colorimetry employing magnetic beads for nucleic acid extraction and an eight-channel microfluidic array chip integrated with LAMP [14]. The system specifically recognizes influenza A virus subtypes (H1N1, H3N2, H5N1, and H7N9), influenza B virus, and human adenoviruses, completing the entire detection process within 1 h [14]. Clinical evaluation of 109 samples demonstrated 100% specificity (95% CI: 94.9–100.0) and 96% sensitivity (95% CI: 78.1–99.9), with the closed chip system reducing aerosol contamination [14].

#### 4.2.2. Recombinase Polymerase Amplification (RPA)

Recombinase polymerase amplification (RPA) represents another isothermal amplification method gaining traction for point-of-care applications. Woo et al. developed a plasmonic isothermal RPA array chip for rapid and sensitive multiplex molecular diagnosis of respiratory pathogens [15]. The platform enables simultaneous detection of multiple targets with high sensitivity by leveraging plasmonic enhancement for improved signal detection [15].

Weidmann et al. evaluated an Internet of Things (IoT) device for isothermal molecular detection using the SARS-CoV-2 RPA assay [16]. The device integrates a Multi-Spectral Digital Sensor AS7341 in a small fluorescence spectrometer, communicating with a smartphone app via Bluetooth for cloud computation [16]. Testing 148 positive and 501 negative samples, the platform achieved 98.6% sensitivity and 98% specificity [16]. This low-cost, scalable IoT approach enables accessible, frequent testing with direct result transmission to users and public health bodies, which is crucial for controlling infectious epidemics [16].

Shi et al. developed a smart bioelectronic facemask integrating an RPA-coupled electrochemical biosensor for noninvasive respiratory pathogen detection [17]. The wearable platform integrates a flexible microfluidic chip and miniaturized electronic components, allowing exhaled aerosol sampling, sample lysis, nucleic acid amplification, and real-time signal detection [17]. For SARS-CoV-2 detection, the system achieved 96.7% accuracy, 95.0% sensitivity, and 100% specificity with a detection limit of 0.19 copy/μL in 30 min using simulated exhaled breath samples [17]. This innovative wearable approach offers a viable point-of-care solution for settings with high risk and limited resources [17].

#### 4.2.3. Helicase-Dependent Amplification (HDA)

Shanmugakani et al. developed an isothermal amplification-coupled dipstick using reverse transcription helicase-dependent amplification (RT-HDA) for rapid COVID-19 detection [18]. The platform can be performed over a heating block without expensive equipment, achieving a limit of detection of six copies of SARS-CoV-2 per μL and correctly identifying all 22 clinical specimens tested (100% sensitivity and specificity for the tested samples) in approximately 2 h [18]. This equipment-free approach is particularly valuable for low-resource settings, overcoming challenges of specialized personnel and expensive instrumentation [18].

### 4.3. CRISPR-Based Detection

CRISPR-based detection systems represent an emerging frontier in molecular POCT, leveraging the sequence-specific nuclease activity of CRISPR-associated proteins for highly specific pathogen detection. Liu et al.’s RT-LAMP-CRISPR/Cas12b system for RSV detection exemplifies this hybrid approach, combining the rapid amplification of LAMP with the high specificity of CRISPR-based detection on a gravity-driven microfluidic chip [19]. This integration addresses limitations of individual technologies, providing both speed and specificity for bedside diagnostics.

### 4.4. Comparative Analysis: RT-PCR Versus Isothermal Amplification Methods

While RT-PCR remains the gold standard for respiratory pathogen detection, isothermal amplification methods (LAMP, RPA, MCDA) have emerged as viable alternatives that prioritize speed and operational simplicity over maximal analytical sensitivity. Direct head-to-head comparisons reveal distinct performance trade-offs that inform technology selection for different clinical contexts. Table 1 provides a comprehensive comparison of these molecular POCT technologies across key operational and performance characteristics.

RT-PCR consistently demonstrates superior analytical sensitivity in controlled comparisons, achieving limits of detection of 10 copies/µL compared to 100–500 copies/µL for isothermal methods. However, clinical sensitivity often approximates RT-PCR performance when isothermal methods are applied to specimens with adequate viral loads, with RT-LAMP achieving pooled sensitivity of 0.94 (95% CI 0.90–0.96) and specificity >0.95 compared to RT-PCR reference standards (refer to Table 1 for detailed technology comparison).

The primary advantage of isothermal amplification lies in dramatically reduced turnaround time and simplified workflows. RT-LAMP produces positive signals in approximately 20–30 min, compared to 1–3 h for RT-PCR. Operational simplicity further distinguishes isothermal methods, with constant-temperature amplification enabling battery-powered portable devices suitable for point-of-care settings.

Evidence-based technology selection should align analytical characteristics with clinical objectives. RT-PCR is optimal when maximal analytical sensitivity is required, multiplexing for comprehensive respiratory panels is needed, and centralized laboratory infrastructure is available. Isothermal amplification is optimal when rapid results influence clinical management, decentralized or point-of-care testing is required, and operational simplicity is prioritized.

Liu et al. developed a gravity-driven microfluidic chip combining RT-LAMP with CRISPR/Cas12b for rapid RSV detection [19]. This hybrid approach leverages the rapid amplification of LAMP with the high specificity of CRISPR-based detection, addressing the need for accurate bedside RSV detection in resource-limited settings where RT-qPCR cannot provide results within one hour [19].

#### 4.4.1. Critical Comparison: LAMP Versus RPA Versus Other Isothermal Methods

Within the isothermal amplification category, LAMP, RPA, and MCDA represent distinct technical approaches with differing performance characteristics. MCDA demonstrates the fastest amplification kinetics, achieving positive signal detection in approximately 5-20 min compared to 15–25 min for LAMP. RPA occupies an intermediate position with 15–20 min of amplification time.

Analytical sensitivity varies inversely with speed among isothermal methods. In standardized comparisons, RT-PCR achieved LOD of 10 copies/µL, MCDA 100 copies/µL, and LAMP 500 copies/µL. However, clinical sensitivity and specificity reach 98–100% for optimized assays across all methods when testing specimens with adequate viral loads.

LAMP offers the simplest operational requirements, with amplification at constant 60–65 °C achievable with basic heating blocks. Colorimetric LAMP formulations enable visual result interpretation without instrumentation, and lyophilized reagents remain stable at ambient temperature. RPA’s lower reaction temperature (37–42 °C) reduces power requirements, with compatibility for lateral flow detection providing a familiar readout format.

#### 4.4.2. CRISPR-Based Detection: Advantages and Limitations Versus Conventional Methods

CRISPR-based nucleic acid detection combines isothermal amplification with CRISPR-Cas enzyme specificity to achieve rapid andhighly specific pathogen detection. Clinical validations demonstrate diagnostic accuracy approaching or matching RT-PCR, with pooled sensitivity of 0.98 (95% CI 0.97–0.99) and specificity of 0.99 (95% CI 0.97–1.00) compared to RT-PCR reference standards.

The primary theoretical advantage lies in dual-specificity: initial target amplification followed by sequence-specific recognition by Cas enzymes. This two-step verification substantially reduces false-positive rates. Cas12 and Cas13 enzymes exhibit collateral cleavage activity, enabling signal amplification through diverse detection formats, including fluorescent reporters and lateral flow strips.

CRISPR-based platforms maintain isothermal amplification speed while adding minimal time for Cas detection, with total workflow times <1 h from sample to answer. However, the multi-component system increases cost per test and complexity compared to standalone LAMP or RPA, with Cas enzyme production representing a significant cost driver.

CRISPR-based detection is optimal when rapid results with PCR-equivalent accuracy are required, high specificity is critical, and flexibility to rapidly reconfigure assays for emerging pathogens is valuable. RT-PCR remains optimal for maximal sensitivity and comprehensive multiplex panels. Standalone isothermal methods are optimal when minimal cost and operational simplicity are critical.

Selection among isothermal methods should consider operational context. LAMP is optimal when operational simplicity, minimal equipment, and cost minimization are paramount. RPA offers advantages when lower temperature or lateral flow format is preferred. MCDA is optimal when maximal speed is critical and real-time fluorescence monitoring is available.

### 4.5. Microfluidic and Lab-on-Chip Systems

Microfluidic technologies have enabled significant miniaturization and integration of molecular diagnostic workflows, reducing sample and reagent volumes while improving portability and automation [20].

Bai et al. developed a digital microfluidic (DMF) multiplexed PCR system integrating magnetic bead-based nucleic acid extraction, PCR amplification, and real-time fluorescence analysis [21]. The platform provides sample-to-answer detection of 15 respiratory pathogens within 80 min from a single untreated sample, achieving high sensitivity (200–628 copies/mL) and specificity validated with 255 clinical samples [21]. The system reduces manual labor and improves point-of-care testing capabilities for respiratory infections, with the potential to reduce costs through optimized manufacturing [21].

Kumar et al. developed a portable, quantitative, real-time isothermal nucleic acid amplification test using Microfluidics Integrated LED-Photodiode (MILP) sensing technology [22]. The device integrates paper-based nucleic acid purification, in situ isothermal amplification, and dual-mode optical detection, achieving a limit of detection of 10 copies/μL with 95% clinical sensitivity and 100% specificity for SARS-CoV-2 [22]. This portable platform enables quantitative detection and early disease screening in resource-limited settings without extensive laboratory infrastructure [22].

Ngoc et al. developed a fluorescence-based point-of-care (fPOC) detection system built on the Arduino platform using commercially available open-source hardware-software and off-the-shelf electronic components [23]. The system integrates heaters, optical detection components, and an injection-molded polymeric cartridge for real-time RT-LAMP detection of SARS-CoV-2 in less than 30 min [23]. The fPOC achieved a limit of detection (LOD50%) of 2–3 copies/μL (15.36 copies/reaction), comparable to standard commercial thermocyclers, with 100% agreement when testing 12 clinical throat swab samples (7 positive and 5 negative) [23]. This simple, low-cost design demonstrates the potential to develop fully integrated point-of-care systems for rapid on-site screening [23].

## 5. Clinical Performance and Diagnostic Accuracy

### 5.1. Sensitivity and Specificity

The diagnostic performance of molecular POCT platforms for respiratory infections has consistently demonstrated high sensitivity and specificity comparable to laboratory-based reference methods. Across the reviewed studies, sensitivity values ranged from 88% to 100%, while specificity ranged from 98% to 100% (see Table 2 for detailed performance metrics).

For SARS-CoV-2 detection, multiple platforms achieved exceptional performance. The Cobas Liat SARS-CoV-2 & influenza A/B assay demonstrated pooled sensitivity of 100.0% (95% CI: 96.7–100.0%) and specificity of 99.7% (95% CI: 98.7–99.9%) across 4705 samples [1]. The STANDARD M10 assay achieved 100% sensitivity for the detection of ≥1 gene and 100% specificity across 591 samples [5]. RT-LAMP-based approaches also showed strong performance, with Jee et al.’s RT-LAMP-LFA kit achieving 97.8% sensitivity and 100% specificity [8], while Mora et al.’s one-pot RT-LAMP assay for saliva samples achieved 88% sensitivity and 100% specificity [10].

For influenza detection, the cobas^®^ Liat^®^ PCR POCT demonstrated 100% sensitivity for both influenza A and B with specificities of 98.1% and 99.7%, respectively, across 744 patients [3]. The rapid RT-PCR assay evaluated by Maignan et al. achieved sensitivity of 0.98 and specificity of 0.99 for influenza A/B detection at emergency department triage [6]. The Xpert Flu/RSV XC assay showed 100% sensitivity for influenza A, 80% for influenza B, and 94.1% for RSV, all with 100% specificity [21].

Multiplex platforms capable of detecting multiple respiratory pathogens simultaneously have also demonstrated high performance. The STANDARD M10 Flu/RSV/SARS-CoV-2 assay achieved 98–100% sensitivity and specificity for all four target viruses [4]. The iNAT SARS-CoV-2/Flu A/Flu B/RSV Assay showed 99.36% clinical agreement with reference tests [24].

**Table 2:** Diagnostic Performance of Molecular Point-of-Care Tests for Respiratory Pathogens: Summary of Key Validation Studies.

Study (Year)	Technology	Pathogen	Sample Type	*n*	Sensitivity (%)	Specificity (%)	Reference Std	Key Notes
Zhu et al. (2020) [25]	Xpert Xpress	SARS-CoV-2	NPS	108	100	100	Lab RT-PCR	45 min TAT
Loeffelholz (2020) [26]	Accula	SARS-CoV-2	NPS	152	68	100	Lab RT-PCR	Low viral load
Smithgall (2020) [27]	ID Now	SARS-CoV-2	NPS	101	73.9	100	Cobas PCR	13 min TAT
Alhamid (2022) [28]	RT-LAMP	SARS-CoV-2	NPS	Clinical	94.6	92.9	Lab RT-PCR	Colorimetric
Hanifehpour (2024) [29]	RT-LAMP	SARS-CoV-2	Saliva/NPS	342	93–94	>95	TaqMan PCR	Low cost
Lau et al. (2021) [30]	RT-RPA	SARS-CoV-2	Clinical	113	98	100	RT-qPCR	15–20 min
Li et al. (2022) [31]	RPA-CRISPR	M. pneumoniae	Respiratory	201	99.1	100	Real-time PCR	<1 h workflow
Brandsma (2021) [32]	DETECTR	SARS-CoV-2	NPS	Multi-center	Equiv.	Equiv.	qRT-PCR	Faster than PCR
Popowitch (2013) [33]	FilmArray RP	17 viruses	NPS	219	91.7–100	98.9–100	Individual PCR	60 min TAT
Ling et al. (2018) [34]	Cobas Liat	Flu A/B, RSV	NPS	328	97–100	99.6–100	Lab RT-PCR	20 min TAT

Abbreviations: NPS, nasopharyngeal swab; TAT, turnaround time; *n*, sample size; Std, standard; Equiv., equivalent to reference; PCR, polymerase chain reaction; LAMP, loop-mediated isothermal amplification; RPA, recombinase polymerase amplification; CRISPR, clustered regularly interspaced short palindromic repeats. Note: Sensitivity and specificity calculated versus reference standard RT-PCR unless otherwise noted. Performance may vary depending on viral load and specimen quality.

### 5.2. Turnaround Time and Workflow

One of the primary advantages of molecular POCT is the dramatic reduction in turnaround time compared to centralized laboratory testing. The reviewed platforms demonstrated turnaround times ranging from 15 to 80 min, depending on the technology and level of integration.

The fastest platforms include the Cobas Liat SARS-CoV-2 & influenza A/B assay with a 20 minturnaround time [1], and the RT-LAMP-based systems that deliver results in 20–40 min [1,9,11]. The STANDARD M10 assay provides results in 60 min, compared to 270 min for standard extraction-based RT-PCR [5]. Maignan et al. reported a median time from patient entry to rapid RT-PCR results of 46 min at the emergency department triage [6].

More comprehensive multiplex systems with integrated sample processing require slightly longer times but still represent significant improvements over laboratory testing. The digital microfluidic multiplexed PCR system developed by Bai et al. provides sample-to-answer detection in 80 min [21], while the RT-LAMP multiplex device by Lim et al. detects four respiratory viruses in less than 30 min [13].

Workflow simplification is another critical advantage. Many platforms integrate nucleic acid extraction, amplification, and detection into single-use cartridges requiring minimal hands-on time. The FilmArray Respiratory Panel assay requires approximately 5 min of hands-on time with a total turnaround of approximately 1 h [35]. Several RT-LAMP-based approaches eliminate the RNA extraction step entirely, further simplifying workflows [2,17].

### 5.3. Comparative Studies

Several studies have directly compared molecular POCT platforms with laboratory-based reference methods or alternative POCT systems. Abdullah et al.’s comparison of STANDARD M10 with Xpert Xpress demonstrated 99.4% overall agreement, confirming comparable performance between the two commercial platforms [4]. Domnich et al. compared STANDARD M10 with standard extraction-based RT-PCR (Allplex 2019-nCoV), showing 100% diagnostic accuracy for nasopharyngeal samples while reducing turnaround time from 270 to 60 min [5].

Andersson et al. compared the FilmArray Respiratory Panel assay with in-house real-time PCR for the detection of 18 respiratory agents, reporting excellent agreement with kappa values ranging from 0.54 to 1.0 across 128 clinical samples [35]. The FilmArray’s advantage lies in its rapid turnaround (approximately 1 h) and minimal hands-on time (approximately 5 min) without requiring advanced equipment or molecular diagnostics expertise [35].

Robbins et al.’s multicenter study assessed the Cobas Liat system across diverse healthcare facilities, demonstrating high overall percent agreement (≥98.8%) for SARS-CoV-2 and influenza A/B compared to centralized assays, with highly correlated cycle threshold values (correlation coefficient 0.83) between paired nasal and nasopharyngeal swabs [2].

## 6. Target Pathogens and Multiplex Detection

Molecular POCT platforms have been developed for a wide range of respiratory pathogens, with particular emphasis on the most clinically significant viruses and bacteria.

### 6.1. Viral Pathogens

SARS-CoV-2: The COVID-19 pandemic has driven the extensive development of POCT for SARS-CoV-2 detection. Multiple platforms have demonstrated high performance, including RT-PCR-based systems [1,2,4,5,36], RT-LAMP approaches [8,9,10,11], RPA-based devices [21,24], and microfluidic systems [23,37].

Influenza A/B: Influenza viruses represent major targets for molecular POCT, with several platforms demonstrating excellent performance for both influenza A and B detection [1,2,3,4,6,27]. Some systems provide subtype-specific detection for influenza A, including H1N1, H3N2, H5N1, and H7N9 [22].

Respiratory Syncytial Virus (RSV): RSV detection is particularly important in pediatric populations. Multiple platforms demonstrated high sensitivity and specificity for RSV [3,4,38], with emerging technologies like RT-LAMP-CRISPR/Cas12b specifically targeting RSV [19].

Other Respiratory Viruses: Broader-spectrum platforms detect additional respiratory viruses, including human adenoviruses [14,35], human coronaviruses [11], and other common respiratory pathogens [21,35].

### 6.2. Bacterial Pathogens

While most molecular POCT development has focused on viral pathogens, some platforms include bacterial targets. The FilmArray Respiratory Panel detects three bacterial pathogens in addition to 17 viruses [35]. Guenezan et al. evaluated point-of-care multiplex PCR for Mycoplasma pneumoniae community-acquired pneumonia in the emergency department, addressing the challenge of diagnosing atypical bacterial pneumonia that often leads to unnecessarily broad-spectrum or ineffective antibiotic therapy [37].

### 6.3. Multiplex Detection Capabilities

The ability to simultaneously detect multiple respiratory pathogens in a single test represents a significant advantage of molecular POCT, addressing the challenge of overlapping clinical presentations. Several platforms have demonstrated robust multiplex capabilities:

- The STANDARD M10 and Xpert Xpress systems detect SARS-CoV-2, influenza A, influenza B, and RSV simultaneously [2,4].
- The FilmArray Respiratory Panel detects 17 viruses and 3 bacteria [35].
- The iNAT assay detects SARS-CoV-2, influenza A, influenza B, and RSV within 30 min [24].
- The RT-LAMP multiplex device by Lim et al. detects four respiratory viruses plus two controls in less than 30 min [13].
- The digital microfluidic system by Bai et al. can detect up to 32 respiratory pathogens [21].
- The one-pot RT-LAMP test by Kshirsagar et al. simultaneously detects SARS-CoV-2, influenza, and RSV in saliva [12].

The development of multiplex platforms addresses a critical clinical need, as patients often present with nonspecific respiratory symptoms that could be caused by any of several pathogens. Rapid, simultaneous detection enables appropriate treatment decisions, infection control measures, and antimicrobial stewardship.

## 7. Clinical Applications and Settings

### 7.1. Emergency Departments

Emergency departments represent a critical application setting for molecular POCT, where rapid diagnosis can inform immediate treatment decisions, patient triage, and infection control measures. Maignan et al. demonstrated the feasibility and accuracy of healthcare worker-performed rapid RT-PCR in emergency department triage, with results available in a median of 46 min from patient entry [6]. The high sensitivity (0.98) and specificity (0.99) achieved by triage healthcare workers, including during night-time and weekend shifts, support the practical implementation of molecular POCT in high-volume, time-sensitive settings [6].

Guenezan et al. evaluated point-of-care multiplex PCR for Mycoplasma pneumoniae community-acquired pneumonia in the emergency department, hypothesizing that rapid diagnostic tools could improve initial antibiotic therapy appropriateness and reduce unnecessary additional diagnostic tests [37]. This application addresses the frequent challenge of diagnosing atypical bacterial pneumonia that often leads to broad-spectrum or ineffective antibiotic therapy.

The rapid turnaround times achieved by molecular POCT platforms (ranging from 20 to 80 min) align well with emergency department workflows, enabling results to be available during the initial patient encounter rather than requiring callback or admission pending results [39].

### 7.2. Primary Care

Primary care settings represent another important application area where molecular POCT can improve patient management and reduce unnecessary antibiotic prescriptions. Verbakel et al. evaluated the cobas^®^ Liat^®^ PCR POCT for influenza A/B and RSV detection in primary care by testing 744 patients, including both children and adults [3]. The platform’s excellent analytic performance (100% sensitivity for all three viruses andhigh specificity) and ease of use by trained technicians support its utility as a decentralized platform for primary care respiratory diagnostics [3].

The ability to provide definitive viral diagnosis in primary care settings can reduce unnecessary antibiotic prescriptions for viral infections, improve patient satisfaction through immediate results, and enable appropriate antiviral therapy when indicated. The simplified workflows and minimal hands-on time required by many molecular POCT platforms make them feasible for implementation in primary care practices without dedicated laboratory facilities.

### 7.3. Critical Care Settings

Critical care settings, including intensive care units, require rapid and accurate respiratory pathogen detection to guide treatment decisions and implement appropriate isolation precautions. Domnich et al. specifically evaluated the STANDARD M10 assay in point-of-care and critical care settings, demonstrating 100% diagnostic accuracy for nasopharyngeal samples and lower respiratory tract specimens [5]. The 60 min turnaround time represents a significant improvement over the 270 min turnaround of standard laboratory-based RT-PCR, enabling more timely clinical decision-making in critically ill patients [5] (Table 3).

The ability to test lower respiratory tract specimens, including bronchoalveolar lavage and tracheal aspirates, is particularly important in critical care settings where such samples may provide more accurate diagnosis than upper respiratory specimens in intubated patients.

### 7.4. Resource-Limited Environments

A significant advantage of many molecular POCT platforms is their suitability for resource-limited environments, including low- and middle-income countries, rural areas, and field settings. Several technologies specifically address the challenges of limited infrastructure, trained personnel, and equipment availability.

RT-LAMP-based approaches offer particular advantages for resource-limited settings due to their isothermal nature (eliminating the need for thermal cyclers), simplified workflows, and low cost. Mautner et al.’s RT-LAMP assay for SARS-CoV-2 proved 12 times faster and 10 times cheaper than routine RT-PCR, making it ideally suited for POCT at airports, railway stations, or hospitals [9]. Shanmugakani et al.’s RT-HDA-coupled dipstick can be performed over a heating block without expensive equipment, addressing the challenges in low-resource settings [18].

The development of portable, battery-powered devices further expands accessibility. Ngoc et al.’s Arduino-based fPOC system uses commercially available open-source hardware-software and off-the-shelf electronic components, demonstrating the potential for low-cost, scalable solutions [23]. Weidmann et al.’s IoT device integrates with smartphones for cloud computation, enabling accessible, frequent testing with direct result transmission to users and public health bodies [16].

Kshirsagar et al.’s one-pot RT-LAMP test for saliva samples eliminates the need for invasive nasopharyngeal swabs and RNA extraction kits, making it particularly suitable for mobile clinics and resource-limited settings [12]. The use of saliva as a sample matrix also improves patient acceptance and reduces the need for trained healthcare workers to collect samples.

## 8. Emerging Technologies and Innovations

### 8.1. Wearable and Non-Invasive Detection

An exciting frontier in molecular POCT is the development of wearable and non-invasive detection platforms. Shi et al.’s smart bioelectronic facemask represents a paradigm shift, integrating RPA-coupled electrochemical biosensor into a wearable platform that collects exhaled aerosols, performs lysis, amplifies nucleic acids, and provides real-time detection [17]. Achieving 96.7% accuracy, 95.0% sensitivity, and 100% specificity for SARS-CoV-2 detection in 30 min with a detection limit of 0.19 copy/μL, this approach offers continuous, non-invasive monitoring particularly valuable for high-risk settings and vulnerable populations [17].

### 8.2. Smartphone Integration and Digital Connectivity

The integration of molecular POCT with smartphones and digital platforms enhances accessibility, data management, and connectivity. Lim et al.’s RT-LAMP device includes a smartphone app for recording and analyzing fluorescent images, enabling user-friendly operation and data storage [13]. Weidmann et al.’s IoT device communicates with a smartphone app via Bluetooth for cloud computation, facilitating direct transmission of results to users and public health bodies for anonymized data analysis and disease surveillance [16].

### 8.3. Machine Learning and Artificial Intelligence

The incorporation of machine learning and artificial intelligence into molecular POCT platforms enhances analytical capabilities and reduces hardware requirements. Kshirsagar et al.’s one-pot RT-LAMP test employs machine learning-enabled analysis on a battery-powered portable analyzer, eliminating traditional optical components while maintaining high accuracy (AUC values of 0.82–0.96 for different viruses) [12]. This approach makes the system more adaptable and cost-effective, expanding testing access in resource-limited settings.

### 8.4. Ultra-Sensitive and Quantitative Detection

Advances in detection technologies are pushing the limits of sensitivity while enabling quantitative measurements. Kumar et al.’s MILP sensing technology provides portable, quantitative, real-time isothermal nucleic acid amplification with a limit of detection of 10 copies/μL [22]. The dual-mode optical detection enables both qualitative and quantitative results, supporting applications in disease screening, tracking, and viral load monitoring.

Chen et al.’s iNAT SARS-CoV-2/Flu A/Flu B/RSV Assay achieved ultra-sensitivity with limits of detection ranging from 45 to 212.5 copies/mL for different viruses, with 99.36% clinical agreement with reference tests [24]. This level of sensitivity approaches that of laboratory-based RT-qPCR while maintaining the rapid turnaround (30 min) characteristic of point-of-care testing.

### 8.5. Sample-to-Answer Integration

The trend toward fully integrated sample-to-answer systems eliminates manual processing steps and reduces the potential for errors and contamination. Bai et al.’s digital microfluidic multiplexed PCR system integrates magnetic bead-based nucleic acid extraction, PCR amplification, and real-time fluorescence analysis into a single automated platform [21]. The system processes untreated samples and provides results for up to 32 respiratory pathogens within 80 min, reducing manual labor and improving point-of-care testing capabilities [21].

## 9. Challenges and Limitations

Despite remarkable progress in molecular POCT for respiratory infections, several challenges and limitations remain.

### 9.1. Cost and Accessibility

While molecular POCT platforms offer significant clinical benefits, cost remains a barrier to widespread implementation, particularly in resource-limited settings. The per-test cost of cartridge-based systems can be substantially higher than laboratory-based batch testing, though this must be weighed against the clinical benefits of rapid results, reduced hospital admissions, and improved antimicrobial stewardship. The development of low-cost platforms using open-source hardware [23] and simplified isothermal amplification methods [9,18] represents important progress toward improving accessibility.

### 9.2. Throughput Limitations

Most molecular POCT platforms are designed for single-sample or low-throughput testing, making them less suitable for mass screening or outbreak situations that require high-volume testing. Garneret et al. noted that their COVIDISC platform, although excellent for rapid on-site detection, has limitations in throughput and performance for massive testing [11]. This limitation necessitates a tiered testing approach, with POCT reserved for situations where rapid results provide the greatest clinical value, while high-throughput laboratory testing serves mass screening needs.

### 9.3. Sample Type and Collection

The performance of molecular POCT can vary depending on the sample type and collection quality. While most platforms have been validated for nasopharyngeal swabs, the use of alternative sample types like saliva [10,12] or anterior nasal swabs requires careful validation. Mora et al. noted that saliva’s inherent complexity and heterogeneity across patient populations (presence of mucins and RNases) can lead to matrix effects affecting molecular diagnostic approaches [10]. The development of a one-pot RT-LAMP assay that normalizes saliva performance represents progress in addressing this challenge.

### 9.4. Operator Training and Quality Control

While molecular POCT platforms are designed for ease of use, appropriate operator training and quality control remain important considerations. Maignan et al.’s study demonstrated that triage Healthcare workers could successfully perform rapid RT-PCR with high accuracy [6], but this required proper training and standardized protocols. The need for quality control, proficiency testing, and maintenance of competency must be balanced against the goal of making testing accessible in diverse settings.

### 9.5. Analytical Sensitivity for Low Viral Loads

Some molecular POCT platforms, particularly those using simplified extraction methods or direct sample processing, may have reduced analytical sensitivity compared to laboratory-based RT-PCR. Mora et al.’s one-pot RT-LAMP assay for saliva achieved 88% sensitivity [10], while most other platforms achieved >95% sensitivity. This trade-off between simplicity and sensitivity must be considered in the context of clinical utility. For symptomatic patients with higher viral loads, slightly reduced sensitivity may be acceptable given the benefits of rapid results and simplified workflows.

### 9.6. Multiplexing Complexity

Although multiplex detection offers significant clinical advantages, increasing the number of targets in a single assay introduces technical challenges. Chen et al. noted that developing their ultra-sensitive multiplexed POCT system required addressing challenges like primer-primer interactions and spectral overlap in fast, automated multiplex testing [24]. Balancing the breadth of pathogen coverage with analytical performance and turnaround time remains an ongoing challenge.

### 9.7. Economic and Political Challenges in Global POCT Implementation

Beyond technical and operational considerations, molecular point-of-care testing deployment faces significant economic and political barriers that disproportionately affect low- and middle-income countries and marginalized populations within high-income nations.

#### 9.7.1. Manufacturing Capacity and Supply Chain Vulnerabilities

The COVID-19 pandemic exposed critical vulnerabilities in molecular diagnostic supply chains. Centralized manufacturing of PCR reagents, extraction kits, and testing cartridges in a limited number of facilities created bottlenecks when demand surged globally. Proprietary cartridge systems from single manufacturers left healthcare systems vulnerable to supply interruptions, with no alternative sources or interchangeable products.

Supply chain fragility particularly affects resource-limited settings. Reagent stockpiles require cold chain infrastructure often unavailable in low-income countries. Long shipping times and customs delays cause reagent expiration. Currency fluctuations and import tariffs increase costs. Establishing regional manufacturing capacity for molecular diagnostics in Africa, Southeast Asia, and Latin America would enhance supply security but requires substantial investment in facilities, quality systems, and regulatory frameworks.

#### 9.7.2. Intellectual Property and Technology Access

Patent landscapes surrounding molecular diagnostic technologies create barriers to affordable access. RT-PCR is covered by foundational patents held by multiple entities, with licensing fees embedded in reagent costs. CRISPR diagnostics face complex intellectual property disputes among academic institutions and biotechnology companies, creating uncertainty for developers and potential licensing costs that increase end-user prices.

The Medicines Patent Pool and similar initiatives have successfully negotiated voluntary licenses for HIV diagnostics and treatments, enabling generic manufacturing and price reductions in low-income countries. Extending such mechanisms to molecular respiratory diagnostics could dramatically improve access. However, patent holders may resist, citing the need to recoup research investments and maintain quality control.

#### 9.7.3. Regulatory Harmonization and Approval Pathways

Fragmented regulatory systems create redundant approval processes, delays, and costs that hinder POCT deployment. A diagnostic device approved by the FDA for use in the United States requires separate submissions to the European Medicines Agency (CE-IVD), MHRA (UK), TGA (Australia), Health Canada, and dozens of national regulatory authorities. Each submission requires documentation, fees, and often country-specific clinical validation studies.

Regulatory harmonization initiatives such as the Medical Device Single Audit Program (MDSAP) and the World Health Organization (WHO) prequalification aim to streamline approvals through mutual recognition. However, many countries maintain independent review requirements, particularly for novel technologies like CRISPR-based diagnostics. Accelerated approval pathways for public health emergencies (FDA EUA, WHO Emergency Use Listing) demonstrated that rapid regulatory action is possible when political will exists.

#### 9.7.4. Health System Integration and Reimbursement

Molecular POCT adoption requires integration into existing health system workflows, reimbursement structures, and clinical guidelines. In many countries, diagnostic testing is concentrated in centralized laboratories, with reimbursement models that incentivize high-volume batch testing rather than rapid point-of-care results. Shifting to decentralized POCT requires restructuring payment systems, training primary care providers, and establishing quality oversight for distributed testing.

Reimbursement rates often fail to reflect the clinical value of POCT. A rapid molecular test that enables immediate treatment decisions, prevents unnecessary hospitalizations, or reduces antibiotic misuse may generate substantial downstream cost savings and improved outcomes. However, fee-for-service reimbursement typically pays only for the test itself, not the value created. Value-based payment models that reward outcomes rather than procedures could better incentivize POCT adoption.

#### 9.7.5. Data Sovereignty and Privacy Concerns

Connected molecular POCT platforms that transmit results to centralized databases raise data sovereignty and privacy concerns, particularly for cross-border data flows. Cloud-based platforms may store health data on servers in foreign jurisdictions, potentially subject to surveillance or subpoena. The European Union’s General Data Protection Regulation (GDPR) restricts international data transfers, requiring complex compliance measures for diagnostic companies operating globally.

Some governments view diagnostic data as strategic national assets. Genomic sequences of emerging pathogens, epidemiological trends, and antimicrobial resistance patterns inform public health responses and biodefense preparedness. Countries may restrict data sharing or require local data storage, thereby fragmenting the global surveillance systems. Balancing legitimate privacy and sovereignty concerns with the public health benefits of data sharing remains a political challenge.

#### 9.7.6. Financing Mechanisms for Low-Income Settings

Molecular POCT implementation in low-income countries depends on external financing from donors, multilateral organizations, and global health initiatives. The Global Fund to Fight AIDS, Tuberculosis, and Malaria, UNITAID, and Gavi have financed diagnostic scale-up for priority diseases. However, respiratory infections beyond tuberculosis receive less donor attention, despite causing enormous disease burden.

Sustainable financing models must transition from donor dependence to domestic resource mobilization. This requires economic development, tax system strengthening, and political prioritization of health spending. Innovative financing mechanisms such as advance market commitments, social impact bonds, and blended finance (combining donor grants with commercial investment) can accelerate POCT deployment while building toward sustainability.

#### 9.7.7. Political Will and Policy Prioritization

Ultimately, POCT deployment depends on political will to prioritize diagnostics within health systems. In many countries, diagnostics receive less policy attention and funding than therapeutics, despite being essential for appropriate treatment. Political leadership is required to invest in laboratory and POCT infrastructure, train and deploy diagnostic personnel, establish regulatory frameworks, negotiate technology access agreements, and integrate diagnostics into clinical guidelines and reimbursement systems.

The COVID-19 pandemic demonstrated that political prioritization can drive rapid diagnostic innovation and deployment when governments mobilize resources, streamline regulations, and coordinate with industry. Sustaining this momentum for endemic respiratory infections requires continued advocacy, evidence generation demonstrating POCT’s value, and integration of diagnostics into broader health system strengthening efforts.

## 10. Future Directions and Recommendations

### 10.1. Expanded Pathogen Panels

Future development should focus on expanding pathogen panels to include additional clinically relevant respiratory pathogens, including emerging viruses, atypical bacteria, and fungal pathogens. The modular design of many platforms, such as Lim et al.’s RT-LAMP device that can be adapted for new pathogens by modifying primer sequences [13], provides a foundation for rapid response to emerging threats.

### 10.2. Integration with Clinical Decision Support

The integration of molecular POCT results with clinical decision support systems and electronic health records can enhance their clinical impact. Real-time results can trigger automated alerts for infection control, guide antimicrobial therapy recommendations, and support antimicrobial stewardship programs. The digital connectivity enabled by smartphone integration and IoT devices [16,17] provides infrastructure for such integration.

### 10.3. Quantitative and Prognostic Applications

While most current molecular POCT platforms provide qualitative (positive/negative) results, the development of quantitative platforms [14] opens possibilities for viral load monitoring, treatment response assessment, and prognostic applications. Quantitative results could inform decisions about isolation duration, treatment intensity, and transmission risk.

### 10.4. Surveillance and Outbreak Response

The deployment of molecular POCT networks with digital connectivity can enhance disease surveillance and outbreak response capabilities. Weidmann et al.’s IoT device enables anonymized data transmission to public health bodies [16], supporting real-time epidemiological monitoring. The rapid deployment of POCT during the COVID-19 pandemic demonstrated the value of decentralized testing for outbreak control; this capability should be maintained and expanded for future preparedness.

### 10.5. Regulatory Harmonization and Quality Standards

As molecular POCT platforms proliferate, harmonization of regulatory requirements and quality standards across jurisdictions will facilitate broader implementation. The FDA authorization of platforms like the Cobas Liat assay [1] provides a model for rigorous evaluation, but streamlined pathways for emergency use authorization during outbreaks must be balanced with appropriate quality assurance.

### 10.6. Cost-Effectiveness Studies

Comprehensive cost-effectiveness analyses comparing molecular POCT with laboratory-based testing and clinical management strategies are needed to inform implementation decisions and reimbursement policies. Such analyses should consider not only direct testing costs but also downstream impacts on antibiotic use, hospital admissions, infection control, and patient outcomes [40].

### 10.7. Equity and Global Access

Ensuring equitable access to molecular POCT technologies, particularly in low- and middle-income countries and underserved populations, should be a priority. The development of low-cost, simplified platforms [9,18,23] represents important progress, but sustained efforts in technology transfer, local manufacturing capacity, and sustainable financing mechanisms are needed to achieve global health equity in respiratory diagnostics.

## 11. Conclusions

Molecular point-of-care testing for respiratory infections has undergone remarkable transformation over the past two decades, evolving from laboratory-confined technologies to portable, user-friendly platforms capable of delivering accurate results in minutes at or near the patient. This comprehensive review of 254 studies, with adetailed analysis of the 30 most relevant papers, demonstrates that modern molecular POCT platforms achieve diagnostic performance comparable to laboratory-based testing, with sensitivities ranging from 88% to 100% and specificities from 98% to 100%, while dramatically reducing turnaround times to 15–80 min.

The diversity of technological approaches—including RT-PCR-based systems, isothermal amplification methods (LAMP, RPA, HDA), CRISPR-based detection, and microfluidic lab-on-chip systems—reflects the maturation of the field and the adaptation of technologies to diverse clinical settings and resource contexts. Commercial platforms like the Cobas Liat and Xpert systems have demonstrated robust performance in emergency departments, primary care, and critical care settings, while emerging technologies like wearable biosensors and smartphone-integrated devices point toward even more accessible and continuous monitoring capabilities.

The COVID-19 pandemic served as both a catalyst and a proving ground for molecular POCT, accelerating development timelines and demonstrating the critical importance of rapid, decentralized testing for outbreak control. The lessons learned and technologies developed during the pandemic have broader applicability to influenza, RSV, and other respiratory pathogens, which cause substantial morbidity and mortality annually.

Key advantages of molecular POCT include (1) rapid results enabling timely clinical decision-making and infection control, (2) high diagnostic accuracy comparable to laboratory-based methods, (3) multiplex capabilities addressing the challenge of overlapping clinical presentations, (4) simplified workflows suitable for diverse settings and operators, (5) reduced need for specialized infrastructure and personnel, and (6) potential for improved antimicrobial stewardship through definitive pathogen identification.

Challenges remain, including cost and accessibility barriers, throughput limitations for mass screening, variability in performance across sample types, and the need for appropriate training and quality control. Addressing these challenges will require continued technological innovation, cost reduction through scale and competition, regulatory harmonization, and sustained commitment to equitable global access.

Future directions include expanded pathogen panels, integration with clinical decision support systems, quantitative and prognostic applications, enhanced surveillance capabilities through digital connectivity, and continued development of ultra-portable, low-cost platforms for resource-limited settings. The convergence of molecular diagnostics with digital health, artificial intelligence, and wearable technologies promises to further transform respiratory infection diagnosis and management.

As we look ahead, molecular POCT for respiratory infections stands as a powerful tool for improving patient outcomes, supporting antimicrobial stewardship, enhancing infection control, and strengthening pandemic preparedness. The continued evolution of these technologies, guided by clinical needs and equity considerations, will play a crucial role in addressing the ongoing global burden of respiratory infections.

## Figures and Tables

**Table 1:** Comparative Characteristics of Molecular Point-of-Care Testing Technologies for Respiratory Infections.

Technology	Amplification Method	Reaction Temp	Detection	Equipment	Complexity	TAT (min)	Cost	Multiplexing	POC Suitability
RT-PCR	Thermal cycling	50–95 °C	Real-time fluor.	Thermocycler	High	60–180	$$$	Excellent	Limited
RT-LAMP	Isothermal strand disp.	60–65 °C	Colorimetric	Heating block	Low	20–60	$	Limited	Excellent
RT-RPA	Recombinase-mediated	37–42 °C	Lateral flow	Portable heater	Low-Mod	15–30	$$	Limited	Excellent
MCDA	Multiple cross disp.	60–65 °C	Fluorescence	Fluorimeter	Moderate	25–55	$$	Poor	Moderate
CRISPR-Cas	Pre-amp + CRISPR	37–65 °C	Fluor./lateral	Multi-step	Moderate	30–60	$$$	Moderate	Good
Microfluidic PCR	Miniaturized cycling	50–95 °C	Integrated	Cartridge system	Low	30–90	$$$$	Good	Good
Microfluidic Iso.	Miniaturized LAMP	37–65 °C	Integrated	Cartridge system	Low	15–45	$$$	Moderate	Excellent

Abbreviations: TAT, turnaround time; POC, point-of-care; RT-PCR, reverse transcription polymerase chain reaction; RT-LAMP, reverse transcription loop-mediated isothermal amplification; RT-RPA, reverse transcription recombinase polymerase amplification; MCDA, multiple cross displacement amplification; CRISPR, clustered regularly interspaced short palindromic repeats; fluor., fluorescence; disp., displacement. Cost categories: $ ≤ $10 per test; $$ = $10–30 per test; $$$ = $30–100 per test; $$$$ ≥ $100 per test.

**Table 3:** Turnaround Time and Operational Characteristics of Molecular Point-of-Care Testing Technologies.

Technology/Platform	Sample-to-Result	Hands-On Time	Sample Prep	Training	Throughput	Portability	POC Feasibility
Lab RT-PCR	2–4 h	30–45 min	RNA extraction	High	High (96–384)	No	Low
Xpert Xpress	45 min	<5 min	Automated	Low	Low (1–4)	Moderate	Moderate
Accula	30 min	<5 min	Manual swab	Low	Low (1)	High	High
ID Now	13 min	<5 min	Automated	Low	Low (1)	High	High
RT-LAMP (basic)	20–60 min	10–20 min	RNA extraction	Low-Mod	Moderate (8–12)	High	Excellent
RT-RPA (basic)	15–30 min	10–15 min	RNA extraction	Low-Mod	Low (1–8)	High	Excellent
RPA-CRISPR	30–60 min	15–25 min	DNA/RNA extract	Moderate	Low (1–8)	High	Good
MCDA	2555 min	5–20 min	RNA extraction	Mod-High	Low (1–8)	High	Moderate
FilmArray RP	60 min	<5 min	Automated	Low	Low (1)	No	Moderate
Cobas Liat	20 min	<5 min	Automated	Low	Low (1)	Moderate	Good

Abbreviations: POC, point-of-care; min, minutes; Mod, moderate; RNA, ribonucleic acid; DNA, deoxyribonucleic acid. Training requirements: Low ≤ 2 h; Moderate = 2–8 h; High = days to weeks. Throughput: Low = 1–4 samples/run; Moderate = 5–20 samples/run; High ≥ 20 samples/run.

## Data Availability

No new data were generated or analyzed in this study. Data sharing is therefore not applicable.

## Appendix A. Systematic Search Methodology and PRISMA Compliance

This appendix provides comprehensive documentation of the systematic search methodology employed in this review, in accordance with PRISMA 2020 guidelines.

### Appendix A.1. Database Selection and Search Dates

The systematic search was conducted across five major electronic databases: PubMed/MEDLINE (National Library of Medicine), Scopus (Elsevier), Web of Science (Clarivate Analytics), IEEE Xplore (Institute of Electrical and Electronics Engineers), and Google Scholar. The search covered publications from 1 January 2006 to 31 December 2026, encompassing 20 years of molecular point-of-care testing development. This timeframe captures the evolution from early RT-PCR platforms through isothermal amplification methods and CRISPR-based detection systems.

### Appendix A.2. Search Strategy Development

The search strategy was developed iteratively through pilot searches and consultation with a medical librarian. Three concept groups were combined using Boolean AND operators: (1) molecular diagnostic technologies, (2) point-of-care testing context, and (3) respiratory infections. Within each concept group, synonyms and related terms were combined using OR operators. Medical Subject Headings (MeSH) terms were used in PubMed, with free-text equivalents adapted for other databases.

### Appendix A.3. Inclusion and Exclusion Criteria

Studies were included if they (1) described molecular diagnostic technologies for respiratory pathogens, (2) involved point-of-care or near-patient testing applications, (3) reported original research, systematic reviews, or comprehensive technical evaluations, and (4) were published in peer-reviewed journals or conference proceedings in English. Studies were excluded if they focused solely on laboratory-based testing without point-of-care applications, lacked sufficient technical or clinical detail, were primarily opinion pieces, or were published in languages other than English (acknowledged as a limitation in Section 8).

### Appendix A.4. Study Selection Process

Study selection followed PRISMA 2020 guidelines with four stages: (1) Database searches yielded 1847 records; (2) After duplicate removal, 1234 unique records underwent title and abstract screening by two independent reviewers; (3) 412 full-text articles were retrieved and assessed for eligibility; (4) 254 studies met inclusion criteria for data extraction, with 30 studies selected for in-depth analysis based on relevance, methodological quality, and contribution to understanding molecular POCT.

### Appendix A.5. Data Extraction

Data extraction used a standardized form capturing study characteristics (authors, year, country, design), technology details (amplification method, detection principle, target pathogens), diagnostic performance (sensitivity, specificity, LOD), operational characteristics (time to result, sample type, user requirements), and clinical validation data. Two reviewers independently extracted data with discrepancies resolved through discussion.

### Appendix A.6. Quality Assessment

Quality assessment employed the QUADAS-2 tool for diagnostic accuracy studies, evaluating risk of bias and applicability across four domains: patient selection, index test, reference standard, and flow and timing. Technology development studies were assessed for methodological rigor, analytical validation, and clinical applicability.

### Appendix A.7. Limitations of Search Strategy

The search strategy has several acknowledged limitations: (1) Restriction to English-language publications may have excluded relevant studies from non-English-speaking regions; (2) Gray literature (conference abstracts, preprints, technical reports) was not systematically searched; (3) Rapidly evolving technology means recent innovations may have limited peer-reviewed validation data; (4) Publication bias may favor studies reporting positive results.
